# Clinical Performance of Bioglass‐Enriched Resin Modified Glass Ionomer in Sealing Moderate Carious Lesions: An 18‐Month Split‐Mouth Clinical Trial

**DOI:** 10.1111/idh.70058

**Published:** 2026-04-14

**Authors:** Juliana Aguiar Costa Mesquita, Ana Carolina Soares Diniz, Mayenne Rabelo Araujo, Etevaldo Matos Maia Filho, Leily Macedo Firoozmand

**Affiliations:** ^1^ Dental Post‐Graduation Program, Department of Dentistry I Federal University of Maranhão (UFMA) São Luís MA Brazil; ^2^ Department of Dentistry I Federal University of Maranhão São Luís MA Brazil; ^3^ Dental Post‐Graduation Program CEUMA University São Luís MA Brazil

**Keywords:** bioactive material, dental caries, glass ionomer cement

## Abstract

Dental materials with the potential for caries progression prevention have been an attractive alternative to minimally invasive treatments.

**Objectives:**

This randomized controlled split‐mouth clinical trial aimed to assess, after 18 months, the retention, restoration quality, and caries progression or arrestment of active moderate carious lesions (International Caries Detection and Assessment System [ICDAS] 3–4) sealed with resin‐modified glass ionomer cement (RMGIC) and 45S5 bioglass‐enriched RMGIC (RMGIC/45S5).

**Methodology:**

A total of 52 molars with occlusal caries (ICDAS 3–4) were selected. For this split‐mouth clinical trial, the permanent molars without mechanical preparation were randomly sealed with RMGIC or RMGIC/45S5. After 18 months, the restorations were compared with the initial treatment (baseline) regarding retention, quality of the remaining material (anatomical shape, marginal adaptation, surface texture, and marginal discoloration), and clinical/radiographic caries progression. The Wilcoxon and Fisher's exact tests were used for data evaluation.

**Results:**

After 18 months, no statistically significant differences were found in the retention and material quality of teeth treated with RMGIC or RMGIC/45S5 (*p* > 0.05). The clinical and radiographic evaluations showed no statistical difference between the materials when considering caries arrestment and progression (*p* > 0.05).

**Conclusions:**

The addition of 45S5 bioglass to RMGIC did not change the retention, restoration quality, and caries progression or arrestment levels after 18 months of follow‐up.

## 1 Introduction

The early diagnosis of incipient carious lesions allows for effective treatments with minimal intervention. Some studies have reported strategies such as the sealing of non‐cavitated carious lesions [1, 2] because sealing these lesions can prevent the diffusion of acids and the loss of dental tissue [1]. However, scarce clinical evidence evaluates the sealing of carious lesions with microcavitated enamel. Thus, the high prevalence of caries, its consequences, and treatment costs reinforce the importance of evaluating carious microcavities in permanent teeth sealed without biomechanical preparations [1].

To assist in the early diagnosis of caries and determination of the appropriate treatment, instruments such as International Caries Detection and Assessment System (ICDAS) have been described in the literature. Within this criterion, ICDAS codes 3 and 4 indicate moderate carious lesions within enamel microcavities and enamel microcavities with dentine shading, respectively [3]. These microcavitations can act as niches that retain biofilms, which limits the effectiveness of fluoride alone and increases the risk of lesion progression, particularly in individuals with high caries activity or who do not adhere well to preventive measures [4]. Sealing or minimally invasively restoring microcavitated lesions has been shown to mechanically protect the affected area, disrupt cariogenic biofilms, and more predictably halt the progression of caries [5, 6].

Sealing of moderate carious lesions (ICDAS 3) with glass ionomer‐based materials in permanent molars appears to be effective in arresting the progression of lesions [7]. However, the risk of material failure due to fracture or retention loss over time can influence caries progression differently [8]. Resin‐modified glass ionomer cement (RMGIC) has good adhesion to tooth tissue, thermic compatibility similar to dentine, and the capacity to release fluoride ions. For these reasons, it has been widely used to treat carious lesions [8, 9].

Dental caries results from the fermentation of dietary carbohydrates by cariogenic bacteria, leading to a decrease in pH and consequently the demineralization of the tooth [10]. In this context, 45S5 bioglass is a material that has been attracting great attention for its remineralizing potential [11, 12]. Laboratory studies have shown that this bioactive glass can release calcium and phosphate ions and has great alkalizing capacity and antimicrobial potential [13]. The association of 45S5 bioglass with RMGIC has been studied in vitro, and a significant improvement in the remineralizing potential of ionomeric materials [14, 15], pH alkalinization in the first hours [9], and an increase in ionomer bioactivity have been observed, which may improve the quality of the restoration [14].

The increased ability of glass ionomer‐based materials to release ions, such as fluoride, calcium, and phosphate, as well as their increased antibacterial potential, could play a key role in promoting the remineralization of dental structures surrounding the material. This ion exchange process helps to form stable chemical bonds with dentine and enamel, which can minimize marginal microleakage and reduce the risk of secondary caries [16, 17]. Despite laboratory studies [9, 13, 18] indicating the benefits of bioglass over RMGIC, the clinical behaviours of these materials for sealing carious lesions remain unknown. Randomized clinical trials help produce scientific evidence demonstrating the long‐term clinical behaviour of bioglass‐enriched RMGIC. The long‐term evaluation of intervention helps visualize the evolution of treatments and determine the best clinical approach [19].

Thus, this split‐mouth randomized clinical trial aimed to compare the sealing behaviours of RMGIC and 45S5 bioglass‐enriched RMGIC (RMGIC/45S5) in moderate carious lesions after 18 months of treatment. The null hypotheses are as follows: (1) there is no clinical and radiographic difference in the progression, stoppage, or regression of moderate carious lesions sealed with the two studied materials and (2) there is no clinical difference in the degree of retention and quality of the sealing performed using RMGIC and RMGIC/45S5.

## 2 Materials and Methods

### 2.1 Ethical aspects and sample selection

Ethical authorization to conduct the study was obtained from the Human Research Ethics Committee of the Federal University of Maranhão, and this study was approved by the Institutional Review Board (IRB), under protocol number (n.2.284.768). The study was also registered in the primary database for clinical studies: The Brazilian Registry for Clinical Studies (ReBEC) with registration code RBR‐389y6y, on May 27, 2021, following the recommendations of the Consolidated Standards of Reporting Trials Statement [20]. Study participants and their parents/guardians signed informed assent and consent forms, respectively.

### 2.2 Sample size calculation and study design

The sample size was calculated using R software (version 4.3.3; R Foundation for Statistical Computing, Vienna, Austria) based on the primary outcome, which was the inhibition of caries progression by clinical and radiographic evaluation after sealing the cavity with RMGIC. In 2016, Wright et al. [21]. observed that under these conditions, resin‐modified GI sealants had a 56% reduction in the risk of developing carious lesions, with an effect size of (OR 0.44; 95% CI, 0.11–1.82), with 80% power and a 5% significance level. Based on these parameters, the required sample size was 64 participants. Due to the split‐mouth design, only 32 participants were needed, and an additional volunteer was enrolled to account for potential losses during the study. A total of 66 teeth were treated, with a dropout rate of approximately 5%–10% (Figure 1).

**FIGURE 1:**
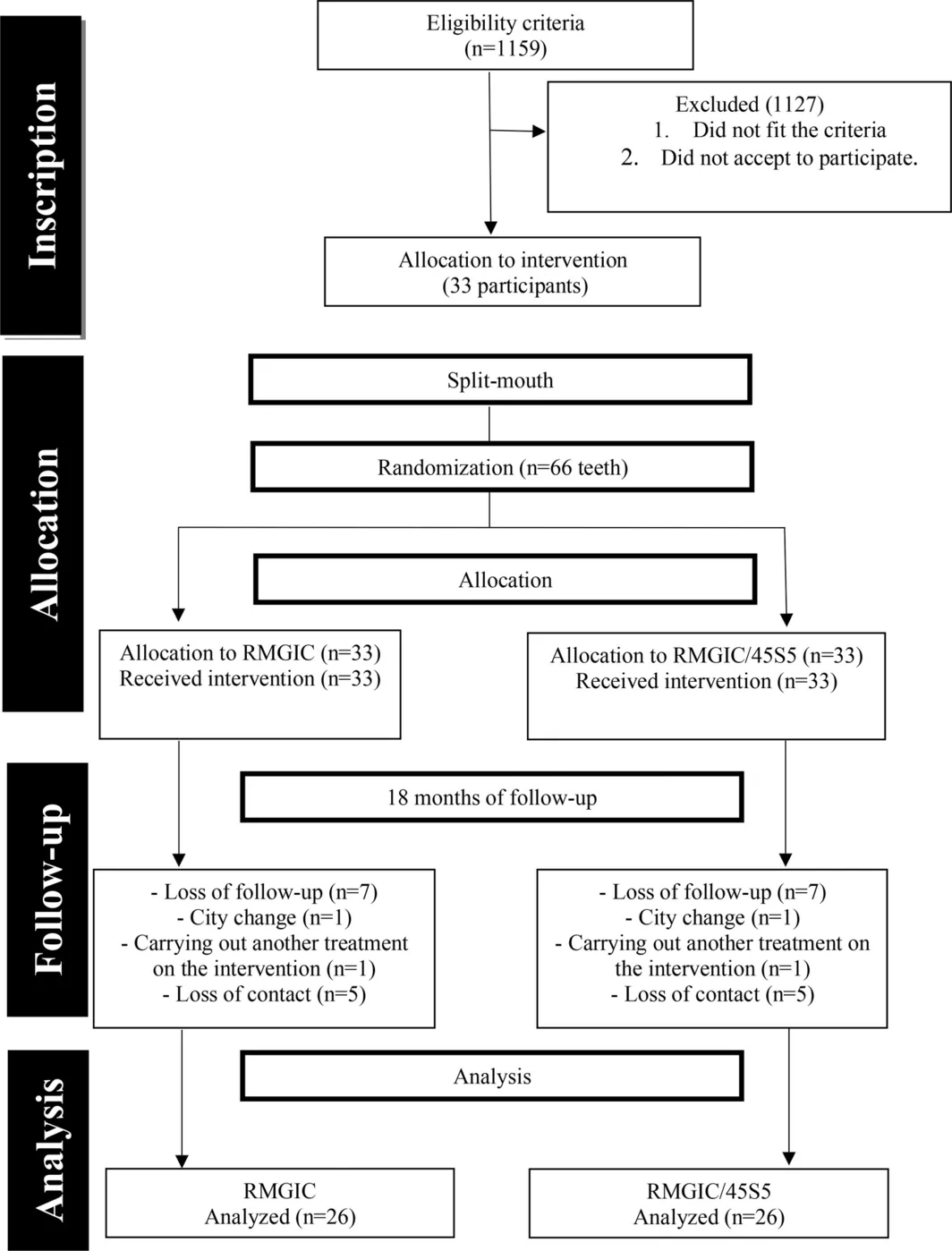
Flowchart of the study carried out.

Data relating to the patients' socio‐nutrition and oral health (such as socio‐nutrition profile, visible plaque index, gingival bleeding index, salivary flow, pH, and buffer capacity) was collected to describe their profiles and contextualize the clinical findings.

The activity strategies were defined in a previous study protocol for this clinical trial, which was conducted and published [22].

### 2.3 Setting and Participants

The target population was schoolchildren between the ages of 8 and 14 from public schools in São Luís, Maranhão, Brazil. Those who volunteered to participate in the study were referred to the Faculty of Dentistry at the Federal University of Maranhão for analysis and treatment.

Participants were eligible if they had good general health; presented at least two permanent molars with carious lesions classified as ICDAS 3 (localized enamel microcavity) or ICDAS 4 (microcavity with underlying dentine shadow), both showing pulp vitality; had no cavitated lesions or restorations on the opposing occlusal surface. Exclusion criteria included the presence of periodontal disease, systemic conditions, allergies or hypersensitivity to any of the materials used, ongoing orthodontic treatment, or a history of dental interventions on the selected teeth before the baseline evaluation.

### 2.4 Characterization of patients to be treated

In this split‐mouth design, each participant contributed two homologous permanent molars (either maxillary or mandibular), with each tooth randomly allocated to one of the two treatment groups (RMGIC or RMGIC/45S5). The selected molars were located in contralateral quadrants to ensure comparable occlusal loading. Only teeth with moderate carious lesions classified as ICDAS 3 or 4, and without cavitation extending to dentine or a history of previous restorations, were included. The cavities were visually and clinically matched based on lesion depth (ICDAS code), location (occlusal), and absence of symptoms, to maintain standardization between the paired teeth.

The randomized triple‐blind split‐mouth model was used in which the participant, the person who administer the treatment, and the evaluator were unaware of the treatment for each of the permanent molars.

#### 2.4.1 Socio‐nutritional and preventive behaviour

After patient selection, a structured questionnaire on socioeconomic and demographic factors was distributed to parents and/or legal guardians. Living conditions, monthly family income, sanitation, and parental education data were collected using the 2020/Brazil Economic Classification Criteria, proposed by the Brazilian Association of Research Companies, which is available at https://www.abep.org/criterio‐brasil. According to socioeconomic class, the participants were categorized into A, B, C, D, and E.

To evaluate the frequency of cariogenic food consumption, the participants answered a Food Frequency Questionnaire [23]. The sum of the daily intake of soft drinks, industrialized juices, drinks with chocolate, chocolate candies, cakes, candies, sweets, and cookies, was dichotomized into 0–3 times a day or > 3 times a day.

#### 2.4.2 Oral health assessment

A previously trained and calibrated examiner evaluated oral hygiene at the beginning of the treatment and after 18 months of follow‐up. A clinical mirror and a Hu Friedy millimetre probe (Hu Friedy, Mgf. Co. Inc., Chicago, USA) with light pressure in the gingival sulcus, parallel to the long axis of the tooth, were used. The probing values obtained in the distobuccal, buccal, mesiobuccal, distopalatal/distolingual, palatine/lingual, and mesiopalatal/mesiolingual areas were registered.

The visible plaque index (VPI) (the presence or absence of plaque was recorded on the four surfaces of each tooth) [24] and the gingival bleeding index (GBI) (the four surfaces of the tooth were evaluated after sliding 0.5 mm of the periodontal probe in the gingival sulcus, being classified as present or absent bleeding) [24] were recorded as clinical parameters.

#### 2.4.3 Salivary evaluation: salivary flow, pH, and buffer capacity

Participants were instructed to properly clean their teeth with floss, brush, and toothpaste and not to eat or ingest liquids for at least 1 h before the collection of the saliva sample.

#### 2.4.4 ‐ Evaluation of salivary flow

Salivation was stimulated by having the patient chew on a small parafilm (PARAFILM “M,” Pechiney Plastic Packing, Chicago, IL, USA) for 5 min. To measure the initial weight, all saliva produced and accumulated in the mouth was stored in a Falcon tube, previously weighed on the Adventurer Precision Digital Scale (OHAUS, Parsippany, NJ, USA). After saliva collection, the tube with saliva was weighed again on the same scale to measure the final weight. These data allowed for obtaining the salivary flow profile of each participant.

#### 2.4.5 ‐ Evaluation of pH and buffering capacity

Salivary pH was estimated using a calibrated digital pH meter (Orion 4 Star, Thermo Scientific, USA). The pH electrode was immersed in the saliva, and the values were recorded. The immersed portion of the electrode was washed with deionized water and cleaned with filter paper before each analysis.

Buffering capacity is a measure of the efficiency of a buffer in resisting pH changes, and this was estimated by the method of Ericsson 195,921. Buffering capacity (β) is expressed as the amount of strong acid or base, in gram equivalents, that must be added to 1 L of the solution to change the pH by one unit. After measuring the initial pH of the saliva, 1 mL of this saliva was added to 3 mL of HCl in an acrylic tube. The mixture was shaken vigorously and allowed to stand for 10 min when the final pH was recorded using a digital pH meter.

Buffering capacity is quantitatively expressed as the ratio of acid added to the change in pH produced, and it was calculated as follows:

$$\text{Buffering capacity}\;\left(\beta \right)=\mathrm{\Delta B}/\mathrm{\Delta pH}$$

In this formula, ΔB = gram equivalent of a strong acid to change the pH of 1 L of buffer solution and ΔpH = pH change caused by the addition of strong acid (final pH − initial pH) [25].

### 2.5 Calibration and randomization

The operator and evaluator, both of whom specialized in restorative dentistry, were trained and calibrated for clinical procedures and analysing patients' oral conditions. The calibration for the use of ICDAS (Kappa = 0.81), Nyvad criteria [26] (Kappa = 0.90) and United States Public Health Service (USPHS) (Kappa = 0.81) index was conducted through theoretical classes, discussion of diagnostic criteria, and photographic and clinical evaluations. The evaluator of the radiographic analysis was trained and calibrated to use ImageJ (Kappa = 0.91).

The teeth to be treated were randomized using an online tool (www.sealedenvelope.com). Allocation details were recorded in opaque envelopes, sequentially numbered, and sealed by a person blinded to the conditions and who did not participate in other steps of the study.

### 2.6 Study intervention: treatment

As previously published in the research protocol [22], a single operator performed all clinical procedures, namely, prophylaxis, absolute isolation, material application (without previous preparation), and occlusal adjustment. Materials were applied using the split‐mouth model. Thus, each participant received two treatments: RMGIC and RMGIC/45S5. The composition and application mode of the products are described in (Table S1).

The bottles of materials used for the treatments were identified with letters A and B so that neither the operator nor the patients were aware of which treatment would be applied to each tooth. The allocation assignment was done using an online tool (www.sealedenvelope.com) revealed during treatments by the assistant, who made the drawn material available to the operator. The revealed treatment was always directed to the right side, and the second to the left side.

Volunteers and their parents/guardians were instructed to follow a preventive program that included oral hygiene, the use of fluoridated toothpaste, and dietary advice and were instructed to return for appointments after 18 months. At the end of the follow‐up, 7 (21.2%) patients were lost to follow‐up, resulting in a final sample of 26 participants and 52 teeth.

### 2.7 Functional and biological property evaluation: 18 months of follow‐up

After the treatment patients were orientated to return periodically. Participants were clinically followed up throughout the entire study period, with regular visits scheduled for monitoring and data collection. The follow‐up protocol aimed to verify sealant integrity, reinforce oral hygiene instructions, and identify any clinical occurrences. During each follow‐up visit, participants were asked about any dental treatments they had received outside of the study protocol. The clinical follow‐ups of this study group were analysed and reported after 1, 3, and 6 months [27].

After 18 months, a new clinical and radiographic evaluation was performed, as well as a new oral health assessment. Diet‐related criteria, socioeconomic status, and the biochemical properties of the saliva were also recorded. Teeth that underwent any external intervention during the follow‐up period were excluded from the final analysis.

The functional and biological properties of the studied materials were directly analysed, considering the USPHS–Modified [28] criteria (anatomical form, marginal adaptation, surface texture, and marginal discoloration, classified as satisfactory when reaching alpha and bravo scores or unsatisfactory with Charlie scores, Table S2). The evaluator was masked as to the treatment performed.

Regarding the functional properties of the restoration, the degrees of retention and fracture of the material were evaluated and classified into 1 (total retention), 2 (partial loss), and 3 (total loss) [29].

### 2.8 Caries activity

#### 2.8.1 Clinical Evaluation

Caries experience was evaluated by comparing the baseline and 18‐month of decayed, missing, and filled teeth (DMFT) [30].

In cases of partial or total material loss, recurrent caries (caries activity and severity in the sealed teeth) were carefully assessed through visual inspection. Visual inspection was used to classify the carious lesions according to the ICDAS [31] and Nyvad [26] criteria. When necessary, the patient was referred for treatment [31].

#### 2.8.2 Radiographic Evaluation

Standardized bitewing radiographs were used to assess caries progression or arrestment. To ensure consistency between the initial and subsequent evaluations, all images were taken with the same radiographic device, exposure parameters, and positioning technique.

An interproximal film (Kodak Insight, Rochester, NY, USA) was positioned similarly to the baseline with the aid of a radiographic positioner. The exposure time was 0.8 s, and the focus–film distance was approximately 25 cm. The films were processed using the temperature/time method.

Images (baseline and after 18 months) were evaluated by a calibrated evaluator who was blinded to the treatment using ImageJ (National Institutes of Health, Bethesda, MD, USA). In each image, the radiolucent area equivalent to the area of the carious lesion was measured three times, obtaining the average of the three measurements as a final result. For the statistical analysis, the injury was categorized into progression (when the radiolucency area was greater than that recorded in the baseline “radiographic progression”) and arrestment (when no difference was found between radiolucency areas “without radiographic progression”) [7].

### 2.9 Statistical Analysis

Descriptive statistics were used to analyse demographic, nutritional, and socioeconomic data to describe the caries risk of the studied group. The Wilcoxon test evaluates oral conditions (DMFT, VPI, and GBI) over 18 months.

Fisher's exact test was used to verify the caries evolution in the studied groups and to determine if an association exists between material type (RMGIC and RMGIC/45S5). The Wilcoxon test was used to evaluate the ICDAS and Nyvad scores at baseline and after 18 months. Additionally, the Wilcoxon test was used to evaluate material retention and quality scores (USPSH) over 18 months.

All analyses were performed using SPSS Statistics for Windows version 26 (IBM Corp., Armonk, NY, USA) at α = 5%.

## 3 Results

### 3.1 Patient Characteristics

A total of 1159 public school volunteers were screened and examined by one experienced examiner using a mouth mirror and a dental explorer. Of this group, 33 volunteers (2.8%) met the inclusion and exclusion criteria and had at least two permanent molars with carious lesions of ICDAS 3 or 4 [26] (Figure 1), making them eligible for analysis. Initially, 33 children, predominantly boys (57.7%), with a mean age of 12.8 years (range 9–16 years) and most with a low monthly family income [27], were included. After 18 months, 26 patients returned for the clinical reassessment visit (Figure 1). Seven children were lost during the follow‐up period because of loss of contact or receiving other treatment; thus, they were not included in the statistical analysis.

To characterize the participant population, the socio‐nutritional profile (Table 1) and oral health‐related variables (Table 2) were explored. These variables included the socio‐nutritional profile, visible plaque index, gingival bleeding index, salivary flow, pH, and buffer capacity. These variables were used to describe the patients' caries risk profiles and contextualize the clinical findings.

**TABLE 1:** Evaluation of the patients at 18 months.

Variables	Individuals *n* (%)
Gender	
Male	15 (57.7%)
Female	11 (42.3%)
Total	26 (100.0%)
Mean age (in years)	12.8
9–12	10 (38.5%)
13–16	16 (61.5%)
Total	26 (100.0%)
Socioeconomic *status CCEB‐ABEP*	
Not answered	5 (19.2%)
A	1 (3.9%)
B	4 (15.4%)
C	11 (42.3%)
D–E	5 (19.2%)
Total	26 (100.0%)
Consumption of foods high in sugar	
1–3×/day	20 (76.9%)
> 3×/day	6 (23.1%)
Total	26 (100.0%)

**TABLE 2:** Oral health status at baseline and after 18 months.

Variables	Individuals		
	*n* (%)		
	Baseline	18 months	*p*
DMFT			
Very low	17 (65.4)	0 (0.0)	0.001
Low	3 (11.5)	1 (3.8)	
Moderated/high/very high	6 (23.1)	25 (96.2)	
Oral health quality (VPI)			
Satisfactory (< 15%)	1 (3.8)	5 (19.2)	0.102
Not satisfactory (≥ 15%)	25 (96.2)	21 (80.8)	
Gingival inflammation (GBI)			
Health (< 15%)	19 (73.1)	2 (7.7)	0.001
Inflammation (≥ 15%)	7 (26.9)	24 (92.3)	

Patients' sex, age, socioeconomic status, and consumption of sugary foods are described in Table 1. Moreover, 23% of the participants had consumed high amounts of sugar‐rich foods (> 3 × /day). Regarding the pattern of consumption of sugary foods, 76.9% of the participants had up to three meals a day, whereas 23.1% had > 3 meals a day (Table 1).

The longitudinal descriptive analysis of oral health‐related variables at baseline and 18‐month parameters (Table 2) supported the clinical relevance of the caries inhibition in the treated teeth during this period. The quality of oral hygiene was also reassessed at 18 months by analysing the number of DMFT, VPI, and GBI (Table 2). At baseline, 65.4% of the participants indicated very low DMFT, 11.5% had low DMFT, and 23.1% had moderate to very high DMFT (Table 2). Of those who indicated very low DMFT at the baseline assessment, 3.8% evolved to low and 96.2% to moderate to very high DMFT at the 18‐month assessment. Of the participants who reported low DMFT at baseline, 100% reported moderate to very high DMFT at the 18‐month assessment, and those who reported moderate to very high DMFT at baseline continued this after 18 months. A significant evolution of DMFT scores was noted over time (*p* < 0.001).

Considering the VPI of patients in the baseline assessment, 3.8% indicated a satisfactory quality of oral hygiene and 96.2% had unsatisfactory quality. After 18 months, 19.2% had a satisfactory quality of oral hygiene, and 80.8% had unsatisfactory quality. No significant difference was observed in the two evaluation periods (*p* = 0.102) (Table 2). As for GBI, in the baseline assessment, 73.1% indicated gingival health, and 26.9% noted gingival inflammation. After 18 months, 7.7% were healthy and 92.3% had inflammation. A significant difference was found in the gingival health in these two evaluation periods (*p* < 0.001) (Table 2).

Treated volunteers showed mean stimulated salivary flow and buffering capacity of 1.57 g/min (±0.79) and 1.50 Meq/pH/mL (±0.55), respectively. These variables all demonstrate the poor oral health that characterizes the presence of caries lesions in high‐risk patients, which can progress significantly.

### 3.2 Dental evaluation: Clinical and radiographic evaluation

The frequencies of each score for the clinical evaluation (sealed, stoppage, and progression) (*p* = 0.052) (Figure 2) and radiographic evaluation (arrestment, moderate progression, and severe progression) (*p* = 0.088) (Figure 3) are visualized. No significant difference was found between the scores for clinical and radiographic (*p* = 0.088) evaluation using RMGIC and RMGIC/45S5.

**FIGURE 2:**
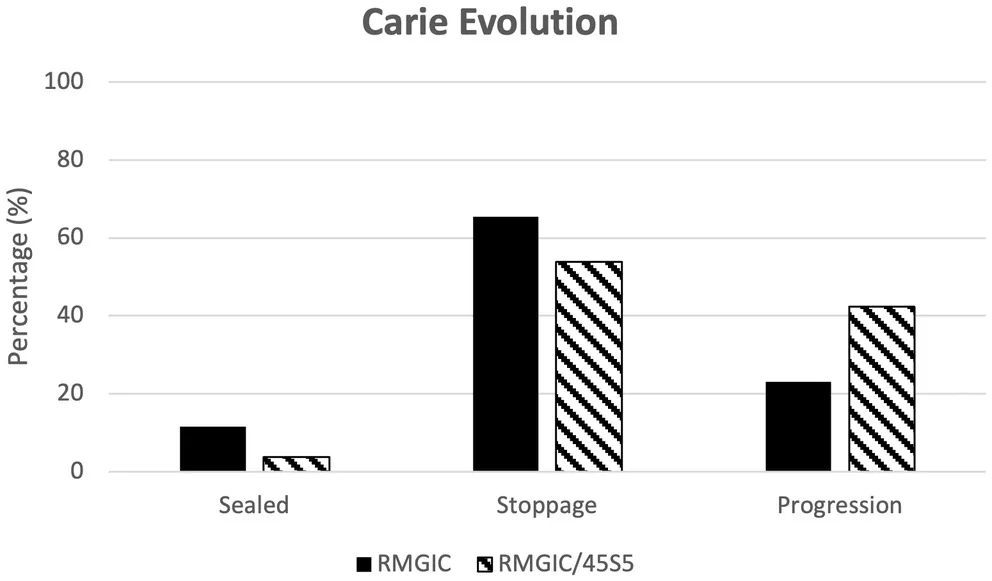
Relative frequency (%) of scores for the clinical assessment of caries evolution (*p* = 0.052).

**FIGURE 3:**
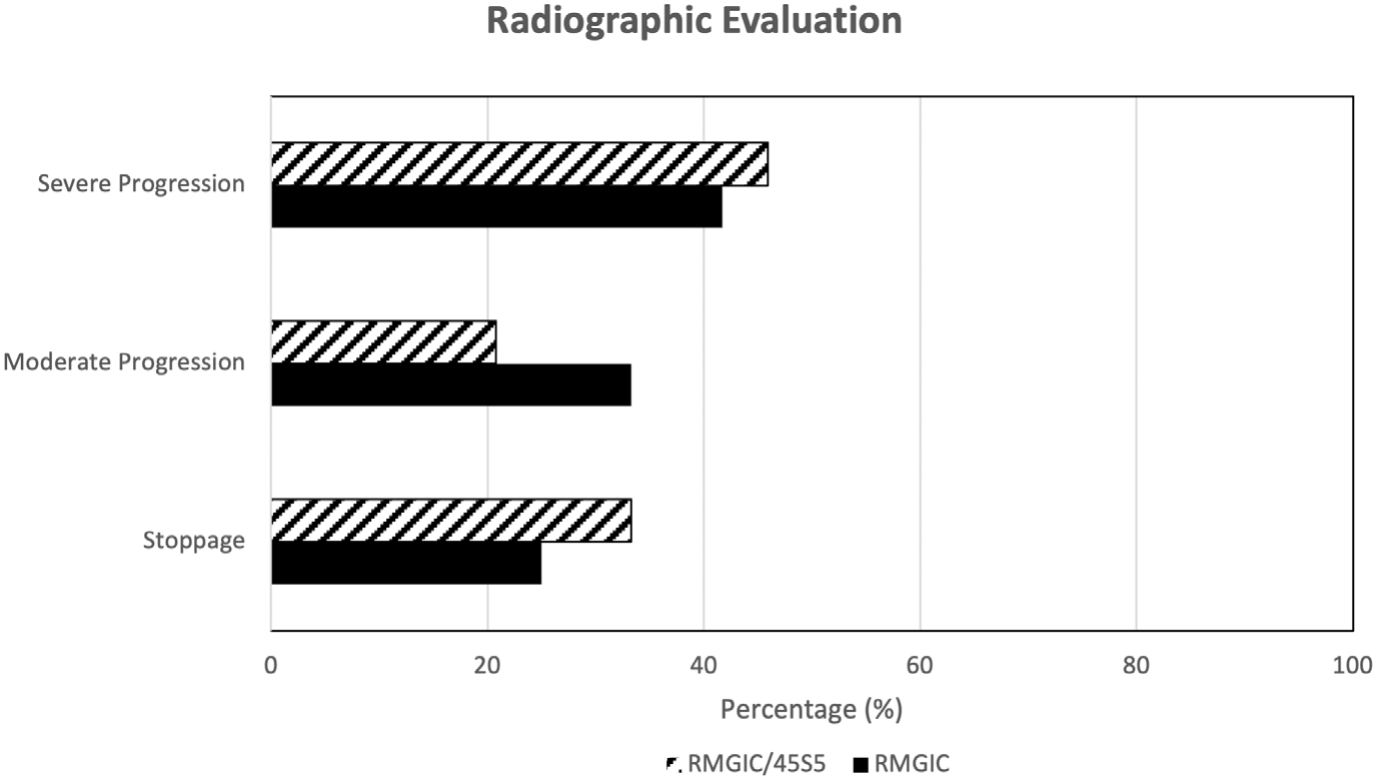
Relative frequency (%) of scores for the radiographic evaluation after 18 months (*p* = 0.088).

#### 3.2.1 Clinical evaluation

The severity and activity of carious lesions were analysed by categorizing the occlusal surfaces using ICDAS and Nyvad criteria, respectively. In this study, 11.5% of the teeth in the RMGIC group and 3.8% in the RMGIC/45S5 group were completely sealed by the material.

As for the ICDAS, in the baseline assessment, the RMGIC group indicated that 3.8% of the participants scored 2, 88.5% scored 3, and 7.7% scored 4, and in the RMGIC/45S5 group, 92.3% of the participants scored 3 and 7.7% scored 4 (*p* > 0.05). In the RMGIC group, the participant who scored 2 in the baseline assessment progressed to a score of 3 in the 18‐month assessment. Of the participants who scored 3 at baseline, 53.8% maintained this score, 19.2% evolved to score 4, and 11.5% and 8% regressed to scores 0 and 2, respectively. Of the participants who scored 4, 3.8% had maintained this score and 3.8% regressed to a score of 3. In the RMGIC/45S5 group, of the participants who scored 3 in the baseline assessment, 3.8% scored 0, 1, and 2; lesion progression stopped in 42.3%; 30.8% evolved to a score of 4; and 7.7% scored 6. Of the participants who scored 4 at baseline, 3.8% maintained this score and 3.8% evolved to a score of 6. No significant difference in the treatment scores was found between RMGIC and RMGIC/45S5 (*p* > 0.05) (Table 3).

**TABLE 3:** Absolute (n) and relative (%) frequencies of the severity (ICDAS) and activity (Nyvad) of carious lesions after 18 months of treatment.

Criteria (18 months)	Scored	RMGIC (%)	RMGIC/45S5 (%)	*p*
ICDAS	Sealed	3 (11.5)	1 (3.8)	0.262
	1	0 (0.0)	1 (3.8)	
	2	1 (3.8)	1 (3.8)	
	3	16 (61.5)	11 (42.3)	
	4	6 (23.1)	9 (34.6)	
	6	0 (0.0)	3 (11.5)	
Nyvad	Sealed	3 (11.5)	1 (3.8)	0.926
	2	1 (3.8)	2 (7.7)	
	3	2 (7.7)	6 (23.1)	
	4	11 (42.3)	10 (38.5)	
	5	9 (34.6)	7 (26.9)	

Relative to Nyvad, the evaluation using this criterion was performed only after 18 months (Table 3). In the sealed group, scores of 2, 3, 4, and 5 were recorded. No significant difference was found in the scores between the RMGIC and RMGIC/45S5 groups (*p* > 0.05).

#### 3.2.2 Radiographic evaluation

After 18 months, a comparative analysis was not possible in two participants because of orthodontic appliances; thus, 24 participants were analysed for each group.

When the lesions were classified as paralysed and progressive, the Chi‐square test verified the lack of difference between the analysed groups (*p* = 0.146). Owing to the varied extensions of the radiolucent lesions, the radiographic evaluation criteria allowed the classification of the carious lesions into three categories for analysis in ImageJ: arrestment in which the measured radiolucent areas at baseline and after 18 months were similar; moderate progression in which the measured radiolucent area after 18 months showed an increase of up to 100% when compared with baseline; and severe progression in which the increase was > 100% of the initial one (Figure 3).

Although the paralysis rate was higher in the RMGIC/45S5 group, this difference was not statistically significant in the radiographic evaluation (*p* > 0.05).

### 3.3 Clinical behaviour of ionomeric cements: retention and material quality

After 18 months, no significant difference (*p* = 0.132) was found in the retention properties of the materials used (Table 4A). The quality of the ionomeric materials (RMGIC and RMGIC/45S5) did not differ statistically concerning the type of treatment performed: anatomical form (*p* = 0.408), marginal adaptation (*p* = 0.257), surface texture (*p* = 0.053), and marginal discoloration (*p* = 0.408) (Table 4B).

**TABLE 4:** Clinical evaluation of materials.

	Total Retention *n* (%)	Partial Retention *n* (%)	Total Loss *n* (%)	TOTAL	*p*
**A. Degree of retention.**					
RMGIC	3 (11.53%)	4 (15.38%)	19 (73.09%)	26	0.132
RMGIC/45S5	(3.84%)	3 (11.53%)	22 (84.63%)	26	

			RMGIC/45S5 *n* (%)	RMGIC *n* (%)	*p*
**B. Clinical assessment (USPHS) of restorations.**					
Anatomical Form	Alpha		1 (3.8%)	2 (7.7%)	0.408
	Beta		1 (3.8%)	3 (11.5%)	
	Charlie		24 (92.4%)	21 (80.8%)	
Marginal Adaptation	Alpha		1 (3.8%)	2 (7.7%)	0.257
	Beta		1 (3.8%)	2 (7.7%)	
	Charlie		24 (92.4%)	22 (84.6%)	
Surface Texture	Alpha		1 (3.8%)	3 (11.5%)	0.053
	Beta		1 (3.8%)	4 (15.4%)	
	Charlie		24 (92.3%)	19 (73.1%)	
Marginal Discoloration	Alpha		2 (7.7%)	2 (7.7%)	0.408
	Beta		0	3 (11.5%)	
	Charlie		24 (92.3%)	21 (80.8%)	

## 4 Discussion

Sealing dental surfaces as a preventive strategy has been reported in clinical studies that have proven its safety and efficiency [2, 32]. The association of 45S5 bioglass in the RMGIC for the treatment of moderate carious lesions (ICDAS 3–4) was the focus of this clinical investigation due to the vast in vitro evidence of its effectiveness. After 18 months, moderate carious lesions (ICDAS 3–4) without biomechanical preparation, treated with conventional RMGIC and RMGIC/45S5, did not show clinical and radiographic differences in the progression and arrest of carious lesions. These materials also did not present a statistical difference in the retention and quality of the restoration. Therefore, null hypotheses (1) and (2) were not rejected.

Clinical evaluation using a split‐mouth model allows the control of oral conditions and reduction of variables. After 18 months in this split‐mouth clinical trial, a follow‐up loss of 21.2% was recorded due to a change in the city of residence, loss of contact, and other treatments in the analysed teeth. This sample loss rate also agreed with the rates of other clinical studies evaluating the sealing of carious lesions at a long‐term follow‐up [19].

Caries risk factors, such as socioeconomic status, sugar consumption, and salivary factors, were also analysed in this study to understand the behaviour of the treated teeth. Most of the study participants had socioeconomic levels C, D, or E, indicating the lowest economic strata according to the CCEB criteria. In agreement with the literature, socioeconomic factors are commonly related to a greater caries experience [33]. Moreover, most patients (77%) consumed sugary food 1–3 times a day. Excessive sugar ingestion causes dental plaque dysbiosis and can result in tooth demineralization [10]. The saliva plays a crucial role in oral homeostasis, helping in the dental remineralization process [25]. Although the salivary flow was considered normal in the study, the ability of the saliva to maintain a constant pH was considered low, which together with excessive sugary food consumption can influence the progression of carious lesions.

The patients had a high risk of caries development, and periodontal conditions showed an unsatisfactory quality of oral hygiene (VPI 96.2% at baseline vs. 80.8% after 18 months) (*p* = 0.102) and an increase in bleeding sites after 18 months (92.3%) when compared with that at baseline (26.9%) (*p* < 0.001). Corroborating this analysis, the number of DMFT increased after 18 months (96.2% at 18 months vs. 23.1% at baseline; *p* > 0.001). Oral hygiene assessment instruments are additional clinical tools to estimate other risk factors because better results are obtained when accompanied by efficient routines. Thus, poor oral hygiene of the study participants corroborates the development of dental caries because the progression of carious lesions in the enamel/dentine depends on the removal of the dental biofilm and the individual's risk of caries development [19].

There is available evidence suggesting that sealants are effective and safe in preventing or arresting the progression of non‐cavitated carious lesions compared to controls without sealants or fluoride varnishes [21]. Therefore, clinical research on microcavitated carious lesions, such as those reported in this study, is needed to provide information about the relative merits of different sealant materials [21]. Considering the aspects related to the preventive effect of the materials, both the clinical and radiographic evaluations indicated that the RMGIC or RMGIC/45S5 did not promote significant differences in the arrest and progression of caries. Some clinical studies have not observed caries progression over time using the RMGIC [7]. Previous laboratory studies believe that 45S5 bioactive glass has a promising remineralization effect on moderate caries [12] and an effective preventive effect on the inhibition of secondary caries in the margins of restorations [34]. RMGIC/45S5 promotes alkalinization and increases the release of calcium, phosphorus, and fluorine ions [18], which can help stop caries progression. Despite the promising laboratory results, given the clinical challenges, the application of RMGIC/45S5 in micro‐cavitated carious lesions did not change the retention and final quality of the restoration.

Clinically, the present study observed that in all teeth in which lesion paralysis was identified, there was partial or complete loss of restoration. The findings of the present study are similar to those of another study suggesting that a restoration protects against caries progression, even when lost over time [35]. It is hypothesized that remnants of the restorative material or ions deposited in dental tissues may have decelerated the progression of carious lesions [36]. Radiographically, in nearly all the teeth with caries progression, the material was totally or partially lost, except for two teeth in the RMGIC group that showed an increase in the radiolucent area even though they were sealed.

In this study, after 18 months of follow‐up, total and partial retention (26.9% and 15.3%, respectively) were observed in the RMGIC and RMGIC/45S5 groups, and no significant difference was found between the materials (*p* = 0.132). Importantly, the treated teeth were not prepared with drills to receive the restorative treatment using only the conditioned retention of the chemical adhesion of the RMGIC to the tooth structure. Similar results were found in other studies using ionomeric materials [32]. Although the literature indicates that partial or total loss of the resin restoration can result in the progression of carious lesions [19], carious lesions do not progress if they are properly sealed [35]. For glass ionomer material, as demonstrated by Mickenautsch and Yengopal (2013) [36], it is important to note that complete retention of occlusal sealants cannot be considered a valid substitute outcome for caries prevention. This is because loss of the material is not necessarily associated with lesion progression, particularly with glass ionomer cement‐based sealants. The glass ionomer may remain at the bottom of the cavity. The systematic review by Gimenez et al. (2023) [37] also reinforces the need for caution when interpreting surrogate outcomes in caries clinical trials.

As for the final quality of the restorations, a higher percentage of the worst evaluation score (Charlie) was found for both materials in all USPHS criteria, with no significant difference for the anatomical form, marginal adaptation, surface texture, and marginal discoloration of the materials studied after 18 months. The final quality of the restorations after the long‐term evaluation was in agreement with those reported in clinical studies that used ionomeric materials, which observed unsatisfactory marginal discoloration and marginal adaptation [38]. Interestingly, ionomeric materials have lower resistance to compression and flexion than other restorative materials, such as composite resin [8].

As recommended in the literature [36] for clinical trials investigating the clinical efficacy of cavity sealing, caries progression was adopted as the primary endpoint and outcome of the study rather than the sealant retention rate. Experimental studies have demonstrated the interesting biological properties of bioactive materials for dental remineralization. One limitation of this study was the need to evaluate a larger patient group over a longer period of time. Nevertheless, preliminary evaluations are important to establish parameters for the clinical behaviour of 45S5 when incorporated into RMGIC. However, more longitudinal clinical studies are needed to confirm the effectiveness of bioglass‐enriched materials in preventing moderate carious lesions.

## 5 Conclusion

After 18 months, the RMGIC/45S5, used to seal moderate carious lesions, resulted in comparable levels of clinical and radiographic caries progression/arrestment. It also had a similar degree of retention and clinical quality to the RMGIC in patients with high‐risk caries.

## 6 Clinical Relevance

After 18 months of evaluation, conventional or bioglass‐enhanced RMGIC shows equivalent levels of retention, restoration quality, and caries progression or arrestment when used to seal active and moderate caries lesions.

### 6.1 Scientific Rationale for Study

Minimally invasive approaches, such as sealing moderate carious lesions with RMGICs, show clinical promise. Bioglass incorporation enhances antibacterial and remineralizing properties in vitro, though clinical evidence remains limited.

### 6.2 Principal Findings

After 18 months of follow‐up, no clinical or radiographic differences were observed between 45S5 bioglass‐enriched RMGIC and conventional RMGIC regarding the progression or arrestment of carious lesions, or the retention or quality of restorations.

### 6.3 Practical Implications

The results support the clinical use of both materials for managing moderate carious lesions. However, more long‐term studies are needed to verify the benefits of bioactive enhancements.

## Funding

This study was financed in part by the Coordenação de Aperfeiçoamento de Pessoal de Nível Superior—Brasil (CAPES)–Finance Code 001 and FAPEMA (Fundação de Amparo a Pesquisa do Maranhão).

## Ethics Statement

All procedures performed in studies involving human participants were in accordance with the ethical standards of the institutional and/or national research committee and with the 1964 Helsinki Declaration and its later amendments or comparable ethical standards. The study was approved by the Institutional Review Board (IRB), under protocol number approval number: (2.284.768).

## Conflicts of Interest

The authors declare no conflicts of interest.

## Supporting information


**Table S1:** Materials used and mode of application.

**Table S2:** Quality of the remaining sealants*.

## Data Availability

The data that support the findings of this study are available from the corresponding author upon reasonable request.
