# Supplementary material for: Clinical Performance of Bioglass‐Enriched Resin Modified Glass Ionomer in Sealing Moderate Carious Lesions: An 18‐Month Split‐Mouth Clinical Trial
Source: Int J Dent Hyg. 2026 Apr 14;24(4):581–91. doi: 10.1111/idh.70058 (PMC13622893; doi:10.1111/idh.70058)
Supplement: Supplementary file 1 [file IDH-24-581-s001.docx]

**Table S1 -** Materials used and mode of application

| **GROUPS** | **PRODUCTS Batch / validity** | **COMPOSITION*** | **MODE OF APPLICATION*** |
| --- | --- | --- | --- |
| RMGIC | Vitro Fil LC (New DFL, Rio de Janeiro, RJ, Brazil)  Batch: 18070450  Validity:04/2020 | **Vitro Fil LC powder:** strontium aluminum fluorine silicate, filler, activators and iron oxide.  **Vitro Fil LC liquid:** 2-hydroxyethyl methacrylate, aqueous solution of polyacrylic and tartaric acids, benzoyl peroxide and camphorquinone.  Vitro Fil Primer: modified methacrylate polyacids, stabilizer, catalyst and ethyl alcohol.  **Natural Glaze:** bisphenol glycidyl methacrylate, triethylene glycols dimethacrylate, 2,6-tert-butylphenol, ethyl urethane, B200P, benzyl dimethyl ketal, camphorquinone and Quantacure EHA. | 1. Perform absolute isolation  2. Condition tooth enamel for 30 seconds with polyacrylic acid, wash and dry well for 30 seconds.  3. Handle the material at the rate of 1 measuring spoon of powder to 2 drops of liquid  4. Divide the powder in half, incorporate the first portion of powder into the liquid and mix for 10 seconds, then mix the remaining portion of powder for a period of 10-15 seconds  5. Apply the material over the area of pits and fissures using an explorer.  6. Photoactivate the material for 20 seconds.  7. Check the occlusion and perform the polishing  8. Apply Natural Glaze and light cure for 20 seconds to seal the surface. |
| RMGIC/45S5 | Vitro Fil LC (New DFL, Rio de Janeiro, RJ, Brasil)  Batch: 18070450  Validity: 04/2020  10% of Bioactive glass SYLC/Osspray Ltd, London, UK | Described above  45% SiO_2_, 24.5% Na_2_O, 24.5% CaO, 6% P_2_O_5_ | 1. The material of the experimental group (RMGIC/45S5) was obtained through the incorporation of 0.5g of the bioactive material (45S5) to the glass ionomer cement modified by Vitro Fil LC resin (New DFL, Rio de Janeiro, RJ, Brazil), after 0.5g of RMGIC has been removed. In this way, 10% of the concentration of the bioactive material was obtained in the RMGIC. After this incorporation, the entire set (RMGIC/45S5) was stirred in a mixer for a period of 5 minutes.  2. Application of the treatment performed in the same way as in the control group. |
