# Supplementary material for: Clinical Performance of Bioglass‐Enriched Resin Modified Glass Ionomer in Sealing Moderate Carious Lesions: An 18‐Month Split‐Mouth Clinical Trial
Source: Int J Dent Hyg. 2026 Apr 14;24(4):581–91. doi: 10.1111/idh.70058 (PMC13622893; doi:10.1111/idh.70058)
Supplement: Supplementary file 2 [file IDH-24-581-s002.docx]

**Table S2** - Quality of the remaining sealants*.

| **Category** | **Classification** | **Criteria** |
| --- | --- | --- |
| **Anatomical form** | Alpha | Continuous |
|  | Bravo | Partially degraded |
|  | Charlie | Completely deteriorated |
| **Marginal adaptation** | Alpha | No visible evidence of a crevice |
|  | Bravo | Visible evidence of a crevice |
|  | Charlie | The explorer penetrates the crevice, and the dentin or base is exposed |
| **Surface texture** | Alpha | No defects |
|  | Bravo | Minimal defects |
|  | Charlie | Severe defects |
| **Marginal discoloration** | Alpha | There is no discoloration anywhere |
|  | Bravo | Discolorations are present but have not penetrated along the margin in a pulpal direction |
|  | Charlie | Discoloration has penetrated along the margin in a pulpal direction |

*Modified USPHS criteria^22^
